# Onset and persistence of person-perceived participation restriction in older adults: a 3-year follow-up study in the general population

**DOI:** 10.1186/1477-7525-6-92

**Published:** 2008-11-05

**Authors:** Ross Wilkie, Elaine Thomas, Sara Mottram, George Peat, Peter Croft

**Affiliations:** 1 Arthritis Research Campaign National Primary Care Centre, Primary Care Sciences, Keele University, Keele, Staffordshire, ST5 5BG, UK

## Abstract

**Background:**

Participation restriction is defined as "problems an individual may experience in involvement in life situations" and refers to the personal and societal consequences of health conditions. There is a growing interest in participation restriction because (i) problems with work or looking after others may be more concerning to individuals than the signs and symptoms of health conditions and (ii) even when poor health persists, participation may still be maintained. The natural history of participation restriction in the general population is unknown and the aim of this report is to describe change in status of person-perceived participation restriction over three years in community-dwelling adults aged 50 years and over.

**Method:**

Prospective cohort study (baseline and 3-year follow-up) using postal questionnaires mailed to a population-based sample of older adults. Responders were included in this study if they completed all items of the Keele Assessment of Participation at baseline (n = 6965). Estimates of onset and persistence of person-perceived participation restriction at 3-year follow-up were calculated for any and for each aspect of life in the sample as a whole, and then by age and gender using attrition re-weighted logistic regression to take account of sample attrition.

**Results:**

In the whole sample of 6965 persons, overall participation status at three years was unchanged in 69%, and changed in 31%. Of 3431 persons with no restriction at baseline, it is estimated that 29.8% (95% confidence interval: 27.6%, 32.0%) would report restriction in at least one aspect of life at 3-year follow-up. Of 3534 persons who had baseline restriction, it is estimated that 68.8% (66.2%, 71.3%) would report continuing restriction in at least one aspect of life after 3 years. Onset and persistence both increased with age, and were most frequently recorded for restricted mobility outside the home.

**Conclusion:**

Although most older persons do not change their overall participation status during a three-year period, change does occur which implies that population approaches to improving participation can be sought. Both onset and persistence of person-perceived participation restriction are more common the older the age-group.

## 

Understanding the impact of health conditions on the lives of older people is important for the development of policies and management strategies to reduce the adverse consequences of ageing in individuals and society [1,2]. The International Classification of Functioning, Disability and Health provides a framework to describe the impact of health conditions on abnormal functioning at three separate levels: anatomical/physiological (impairment); individual (activity limitation); and societal (participation restriction) [3].

Our knowledge of the natural history of these three levels and of the underlying health conditions, is uneven. The prevalence and incidence of several health conditions and impairments (e.g. cardiovascular disease [4]; osteoarthritis [5]; cognitive impairment [6]; diabetes [7]) increase strongly with age. The impact of these on activity limitation has been previously investigated and, although the prevalence and incidence of activity limitations, also increase with age [8], recent studies suggest a dynamic process, often involving multiple episodes, recovery from single episodes, and episode recurrence [9-16]. Much less is known about the pattern of participation restriction, which is defined as "problems an individual may experience in involvement in life situations" and refers to the personal and societal consequences of health conditions [3]. There is a growing interest in such consequences and the influence of environmental factors on them [17-19]. Participation in life situations, such as looking after others and work, as well as psychological well-being [20], may be of more concern to individuals than the underlying impairments and activity limitation [21].

We have previously reported from cross-sectional data that person-perceived participation restriction is common in adults aged 50 years and over, increases with age and female gender, and most commonly affects mobility outside the home [22]. Incidence and recovery rates of participation restriction in the general population are not known. Such information would help to establish the extent to which older adults maintain or regain their desired levels of function in everyday life – despite an increasing frequency of health conditions, impairments and activity limitations – by adaptation [23,24], the use of aids [25-27], assistance from others [28,29] and changes in perception [30,31]. The aim of this prospective analysis was to describe the onset and persistence of person-perceived participation restriction in a population sample of older people, over a period of three years.

## Methods

The design was a prospective cohort study, based on postal surveys at baseline (April 2002) and 3-year follow-up (April 2005), in an older adult population. Ethical approval for the study was obtained from the North Staffordshire Local Research Ethics Committee.

### Study design and participants

Details of the recruitment survey have been presented previously [32,33]. In summary, the population registers of three general practices from the North Staffordshire Primary Care Research Consortium were used as a population sampling frame to mail 11230 adults aged 50 years and over with a postal questionnaire about health and lifestyle matters. 7259 adults returned questionnaires in which all items in one section (Keele Assessment of Participation (KAP)[34]) had been completed (overall response = 71.3%) (April 2002). This group as a whole were very similar in general health, age, gender and marital status to UK norms, suggesting that there was no substantial non-response bias [22]. 5032 gave written permission for further contact and were eligible for the follow-up study (Figure) 1.

**Figure 1:**
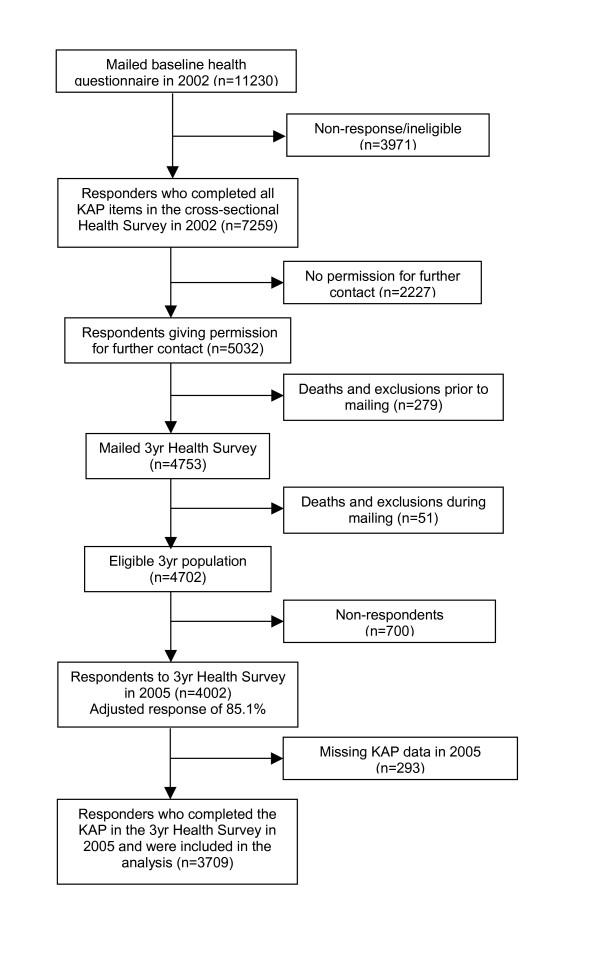
Flow diagram of participants.

There were 3709 fully completed questionnaires returned at 3 years, giving a follow-up response of 73.7% among persons who were eligible and had consented to the follow-up and representing 51% of the original responding population at baseline. Those lost to follow-up include 294 deaths, 12 departures and 24 exclusions because of terminal illness or severe psychiatric illness; non-responders among the remaining persons for follow-up included 72 people who declined to participate, 29 people who stated ill health as the reason for not completing the questionnaire, 599 people from whom no response was received and 293 returned questionnaires with incomplete KAP items. The study population for the analysis of observed data thus consisted of the 3709 persons who returned fully completed questionnaires on both occasions. Estimated data was calculated for adults who completed the KAP at both time points and those who completed the KAP at baseline and were lost to follow-up (this is defined as those who did not consent to follow-up, non-responders at follow-up, exclusions and those who did not complete the KAP (n = 6965)).

### Data collection

The baseline and follow-up questionnaires collected information on demographic, socioeconomic and general health characteristics, including the Short Form-12 instrument (SF-12) [35]. Participation restriction was measured by the KAP [34]. This short self-complete instrument is designed to measure participation from the perspective of the individual in 11 aspects of life mapped to domains and sub-domains of the ICF: mobility within the home, mobility outside the home, self-care, looking after belongings, looking after the home, looking after dependents, interpersonal interaction, managing money, work, education, and social activities. Items are phrased to capture performance ("I have"), individual judgement, and the nature and timeliness of participation ("as and when I have wanted"). Responses are on a five point ordinal scale (All/Most/Some/A little/None of the time) and responders were considered restricted in an aspect of life if they did not participate in it "as and when they wanted" for "all" or "most of the time". The number of aspects of life, where responders indicated participation restriction, was calculated for both time points and categorised to (i) any restriction (participation restriction in at least one aspect of life) and (ii) minimal (1–2 restricted aspects of life) or moderate/substantial (3–11 restrictions (moderate and substantial were combined due to small numbers in the substantial category for some items (e.g. work)). The reliability and validity of the KAP have been established as adequate for providing estimates of perceived participation restriction in population studies [34].

### Statistical analysis

Data recorded in the baseline survey was used to assess the effect of (i) loss to follow-up and (ii) death prior to follow-up, by examining differences in baseline demographics (age, gender), socio-economic status (occupation, educational attainment), general health (physical and mental SF-12 scores) and participation restriction. Percentages and means were used to compare groups with different follow-up outcomes.

The following analysis was carried out on the observed data, i.e. on those completing the 3-year follow-up, and to assess the effects of loss to follow-up, we repeated the onset and persistence analyses assuming that those lost at follow-up had the same likelihood of participation restriction (examining onset and persistence separately), within strata defined by age, gender, educational attainment, occupational class and general health (using SF-12 physical and mental health scores), as those who did complete the three year follow-up questionnaire. These six strata-defining variables were selected a priori as they were used to describe the differences between responders and those lost to follow-up. These estimates were calculated using attrition re-weighted logistic regression [36] performed in Stata 9.2.

The frequencies of three-year onset of participation restriction (defined as moving from no restriction at baseline to any restriction at three year follow-up) and three-year persistence (any restriction at both baseline and follow-up) were calculated overall, and by age and gender, and summarized using percentages.

For those with three-year persistence, the proportion that reported (i) the same number and same restrictions at baseline and follow-up, (ii) the same number but different restrictions at follow-up, (iii) a lower number at follow-up and (iv) a greater number at follow-up, were calculated.

Onset of restriction for each of the 11 aspects of life was calculated as the proportion of those not restricted in that aspect of life at baseline who reported restriction in that aspect at 3 years, regardless of whether the individual reported restriction in other items at baseline or three year follow-up. This analysis was also conducted stratified by age group and gender.

In individuals with participation restriction at baseline, estimates of persistence within each of the 11 aspects of life were calculated overall, and by age and gender. Persistence was calculated as those with restriction in an aspect of life at both baseline and follow-up.

To examine the link between onset of restriction in each aspect of life and amount of restriction at baseline (0, 1–2, 3–11), we used logistic regression, unadjusted, adjusted for gender and, separately, adjusted for age (10 year bands). Associations are presented as odds ratios, with 95% confidence intervals.

## Results

### Baseline differences between those who completed all KAP items at three-year follow-up, those lost to follow-up and those who died before follow-up

Persons who completed all KAP items (n = 3709) at 3-year follow-up, compared with the 3256 persons lost to follow-up, had higher baseline levels of physical health (SF-12 physical health score: 42.3 v 39.9) (Table 1) and mental health (SF-12 mental health score: 49.8 v 48.3), and were more likely to be younger (aged 50 to 69: 72.7% v 55.4%), have gone on to further education (41.1% v 25.6%) and be classified as having a non-manual occupation (48.7% v 34.7%). They were also less likely to report participation restriction (any restriction: 44.7% v 57.6%). In turn, compared with 294 persons who died between baseline and follow-up, persons lost to follow-up had higher levels of physical health (39.9 v 32.1), and mental health (48.3 v 44.3), and were more likely to be younger (aged 50 to 69: 55.4% v 27.9%). They were less likely to report participation restriction (any restriction: 57.8% v 76.5%).

**Table 1:** Baseline characteristics of those with completed KAP, lost and dead at follow-up

	Completed KAP at follow-up (n = 3709) No (%)	Lost to follow-up (n = 3256) No (%)	Dead at follow-up (n = 294) No. (%)
**Age (years)**			
50–59	1432 (38.6)	936 (28.7)	22 (7.5)
60–69	1264 (34.1)	871 (26.7)	60 (20.4)
70–79	801 (21.6)	931 (28.6)	115 (39.1)
80+	212 (5.7)	518 (15.9)	97 (33.0)

**Gender**			
Male	1645 (44.4)	1385 (42.5)	172 (58.5)
Female	2064 (55.7)	1871 (57.5)	122 (41.5)

Educational attainment*			
Further education	1514 (41.1)	822 (25.6)	86 (29.5)
School education only	2174 (59.0)	2392 (74.4)	206 (70.6)

Occupational class*			
Non-manual	1718 (48.7)	1001 (34.7)	123 (44.9)
Manual	1809 (51.3)	1887 (65.3)	151 (55.1)

**Participation restriction**			
Any (≥1 aspect)	1660 (44.8)	1874 (57.5)	225 (76.5)
Minimal (1–3 aspects)	1250 (33.7)	1322 (40.6)	125 (42.5)
Moderate (4–6 aspects)	308 (8.3)	433 (13.3)	81 (27.6)
Substantial (7–11 aspects)	102 (2.8)	119 (3.7)	19 (6.5)

SF-12 Physical health score*			
Mean (SD)	42.3 (12.3)	39.9 (12.5)	32.1 (11.0)

SF-12 Mental health score*			
Mean (SD)	49.8 (11.1)	48.3 (11.2)	44.3 (11.7)

* – is subject to missing data

There was little difference between the attrition re-weighted estimates in any of the onset and persistence analyses. Therefore, all estimates presented here are for the attrition re-weighted analyses. The observed data are available in additional file 1 Observed onset and persistence of restriction in any and each aspect of life at three years.

### Onset and persistence of any restriction

Among the whole study population of this analysis (n = 6965), 69% did not change their overall participation status, whilst 31% either developed or recovered from participation restriction. The estimated proportion of 3431 participants with no restriction at baseline, who would report restriction at three-year follow-up was 29.8% (95% confidence interval: 27.6%, 32.0%) (Table 2). This estimated proportion was similar in women and men (29.0% (26.1%, 32.1%) v 30.6% (27.6%, 33.8%), and increased with age in both genders, although this trend was more pronounced in women. Of those with no restriction at baseline, 26.5% (24.4%, 28.6%) would indicate restriction in one to three items and 3.3% (2.5%, 4.3%) in four to eleven items, at three-year follow-up.

**Table 2:** Estimates of the onset and persistence of any restriction at three-year follow-up in older adults who completed the KAP at three year follow-up and those lost at follow-up; overall and stratified by age and gender

	**Estimated onset of any restriction**	**Estimated persistence of any restriction**
	**% (95% CI)**	**% (95% CI)**
**Overall**	29.8 (27.6, 32.0)	68.8 (66.2, 71.3)

**Gender**		
Females	29.0 (26.1, 32.1)	72.4 (69.1, 75.4)
Males	30.6 (27.6, 33.8)	63.9 (59.8, 67.8)

**Age group**		
Overall		
50–59 years	26.4 (23.4, 29.6)	62.8 (58.5, 66.9)
60–69 years	26.7 (23.4, 30.4)	63.6 (58.9, 68.1)
70–79 years	36.9 (31.8, 42.3)	74.7 (69.5, 79.2)
80+ yrs	48.0 (35.5, 60.8)	86.3 (77.7, 91.2)
		
Females		
50–59 years	25.4 (21.4, 29.6)	66.3 (60.6, 71.5)
60–69 years	26.5 (22.0, 31.6)	69.3 (63.2, 74.9)
70–79 years	35.1 (28.1, 42.8)	75.0 (68.4, 80.6)
80+ yrs	51.4 (33.2, 69.2)	89.6 (80.7, 94.7)
		
Males		
50–59 years	27.4 (23.0, 32.3)	58.4 (51.7, 64.7)
60–69 years	27.0 (22.3, 32.2)	56.8 (49.6, 63.7)
70–79 years	38.8 (31.7, 46.4)	74.2 (65.5, 81.4)
80+ yrs	45.1 (28.4, 63.0)	79.7 (59.6, 91.3)

The estimated proportion of 3534 participants with any restriction at baseline, who would report restriction at three-year follow-up was 68.8% (66.2%, 71.3%). The proportion with persistence of restriction was higher in women than men (72.4% (69.1%, 75.4%) v 63.9% (59.8%, 67.8%), and increased with age in both genders, although more so in women than men.

However the overall picture in the persistent group hides variation in the change in status of individual participation items. Of those who would indicate three-year persistence, 12.3% (10.3%, 14.6%) would indicate the same restrictions at both time points, 20.1% (17.5%, 22.9%) would indicate the same number of restrictions but different areas would be restricted, 35.1% (32.0%, 38.4%) would indicate a lower number of restrictions and 33.2% (29.5%, 35.8%) would indicate a greater number of restrictions at follow-up.

### Onset and persistence for individual aspects of restriction

Onset of restriction at 3 years in individual aspects of life ranged from 3.0% (2.5%, 3.7%) (work) to 12.2% (10.9%, 13.6%) (mobility outside the home) (Table 3). Onset increased with age for 8 aspects of life and decreased with age for the remaining 3 aspects of life (looking after dependents, work, education). Onset was more frequent in women than in men for two aspects of life (mobility outside the home, interpersonal interaction) (data not shown).

**Table 3:** Estimates of onset and persistence of participation restriction in individual aspects of life at three years in older adults who completed the KAP at three year follow-up and those lost at follow-up

	**Mobility** ** within** ** the** ** home** **% ** **(95% CI)**	**Mobility** ** outside ** **the** ** home** **%** ** (95% CI)**	**Self-** **care** **% ** **(95% CI)**	**Looking** ** after** ** the** ** home** **% ** **(95% CI)**	**Looking** ** after** ** belongings** **%** ** (95% CI)**	**Looking** ** after** ** dependents** **% ** **(95% CI)**	**Interpersonal** ** interaction** **% ** **(95% CI)**	**Managing** ** money** **% ** **(95% CI)**	**Work** **% ** **(95% CI)**	**Education** **% ** **(95% CI)**	**Social** ** activities** **% ** **(95% CI)**
Onset of restriction											
											
Overall	6.1 (5.2, 7.2)	12.2 (10.9, 13.6)	6.1 (5.2, 7.1)	8.6 (7.6, 9.9)	5.7 (4.9, 6.8)	3.9 (3.3, 4.7)	8.1 (7.1, 9.2)	10.6 (9.4, 11.8)	3.0 (2.5, 3.7)	3.4 (2.8, 4.1)	10.1 (9.0, 11.3)
50–59 years	3.6 (2.7, 4.9)	7.1 (5.7, 8.8)	4.0 (3.0, 5.3)	7.2 (5.8, 9.0)	5.0 (3.8, 6.4)	4.6 (3.5, 6.0)	5.9 (4.6, 7.4)	8.7 (7.2, 10.6)	3.5 (2.6, 4.7)	5.8 (4.6, 7.2)	9.3 (7.7, 11.1)
60–69 years	4.6 (3.4, 6.2)	9.2 (7.4, 11.3)	5.3 (4.0, 6.9)	6.6 (5.1, 8.4)	4.6 (3.4, 6.3)	4.2 (3.1, 5.6)	6.1 (4.8, 7.9)	10.8 (9.0, 13.0)	3.4 (2.4, 4.7)	2.8 (2.0, 4.0)	8.8 (7.1, 10.8)
70–79 years	8.6 (6.4, 11.3)	20.0 (16.6, 24.0)	7.1 (5.2, 9.7)	11.7 (9.1, 14.9)	6.9 (5.0, 9.6)	3.1 (1.9, 5.0)	11.0 (8.6, 14.1)	12.7 (10.1, 16.0)	2.7 (1.6, 4.3)	1.7 (1.0, 2.9)	12.6 (9.9, 15.7)
80+ years	18.6 (12.3, 27.1)	35.9 (26.5, 46.6)	18.2 (12.1, 26.4)	15.3 (9.6, 23.5)	11.0 (6.5, 18.2)	2.1 (0.7, 6.3)	19.8 (13.6, 27.9)	11.2 (6.6, 18.3)	0.3 (0.1, 2.3)	0.8 (0.2, 3.2)	11.2 (6.9, 17.7)

Persistence of restriction											
Overall	44.7 (38.8, 50.7)	63.7 (59.6, 67.6)	39.9 (33.2, 47.0)	49.5 (44.3, 54.8)	41.4 (35.5, 47.6)	21.5 (16.0, 28.3)	50.7 (45.2, 56.1)	47.0 (42.1, 52.0)	10.9 (6.7, 17.3)	18.4 (13.1, 25.2)	32.6 (28.2, 37.3)

Onset of restriction in individual aspects of life was more frequent in those who had restriction in other aspects of life at baseline (Table 4). These relationships were not affected by adjustment for age or for gender. The association with onset was stronger as baseline restriction increased for all aspects of life, other than for looking after dependents and for work and education.

**Table 4:** Estimated associations between the onset of restriction in individual aspects of life and amount of restriction at baseline, unadjusted, adjusted for gender and adjusted for age; Odds ratios and 95% confidence intervals

	Unadjusted OR* (95% CI)^†^	Gender adjusted Adj OR (95% CI)	Age adjusted Adj OR (95% CI)
Mobility within the home			
1–2	2.67 (1.79, 3.97)	2.68 (1.80, 3.98)	2.40 (1.60, 3.60)
3+	7.53 (4.76, 11.91)	7.61 (4.81, 12.05)	6.52 (4.10, 10.35)

Mobility outside the home			
1–2	1.91 (1.45, 2.52)	1.91 (1.45, 2.51)	1.86 (1.40, 2.46)
3+	3.24 (1.94, 5.40)	3.20 (1.93, 5.32)	3.30 (1.92, 5.66)

Self-care			
1–2	2.42 (1.60, 3.66)	2.42 (1.60, 3.66)	2.20 (1.45, 3.34)
3+	8.12 (5.30, 12.46)	8.16 (5.34, 12.48)	7.40 (4.52, 11.36)

Looking after the home			
1–2	2.19 (1.59, 3.03)	2.19 (1.59, 3.02)	2.07 (1.49, 2.88)
3+	5.99 (3.88, 9.26)	5.98 (3.87, 9.23)	5.53 (3.56, 8.61)

Looking after belongings			
1–2	2.31 (1.52, 3.49)	2.29 (1.52, 3.47)	2.21 (1.46, 3.37)
3+	7.07 (4.48, 11.14)	7.00 (4.46, 1.00)	6.54 (4.05, 10.58)

Looking after dependents			
1–2	1.03 (0.63, 1.66)	1.02 (0.64, 1.65)	1.10 (0.68, 1.78)
3+	3.06 (1.95, 4.81)	3.03 (1.92, 4.78)	3.54 (2.25, 5.55)

Interpersonal interaction			
1–2	2.67 (1.92, 3.70)	2.65 (1.91, 3.67)	2.46 (1.77, 3.42)
3+	5.67 (3.72, 8.65)	5.58 (3.67, 8.48)	5.07 (3.29, 7.86)

Managing money			
1–2	1.64 (1.21, 2.21)	1.63 (1.21, 2.20)	1.62 (1.20, 2.18)
3+	2.08 (1.44, 3.00)	2.05 (1.42, 2.96)	2.03 (1.40, 2.95)

Work			
1–2	1.24 (0.78, 1.97)	1.23 (0.78, 1.95)	1.33 (0.83, 2.13)
3+	1.10 (0.60, 2.03)	1.08 (0.59, 1.98)	1.25 (0.67, 2.32)

Education			
1–2	1.14 (0.76, 1.72)	1.14 (0.76, 1.73)	1.31 (0.87, 2.00)
3+	0.57 (0.30, 1.10)	0.57 (0.30, 1.11)	0.69 (0.35, 1.34)

Social activities			
1–2	1.53 (1.14, 2.06)	1.53 (1.14, 2.06)	1.50 (1.11, 2.02)
3+	2.07 (1.41, 3.04)	2.08 (1.41, 3.05)	1.99 (1.35, 2.94)

* Odds ratio; ^† ^95 percent confidence interval

Persistence of restriction in individual aspects of life, among persons with baseline participation restriction, was highest for mobility outside the home (63.7% (59.6%, 67.6%)) and was lowest for work (10.9% (6.7%, 17.3%)) (Table 3). Persistence increased with age for one aspect of life (mobility outside the home) and decreased with age in one aspect (looking after dependants). There was no relationship between persistence and age for the remaining nine aspects of life. Persistence was greater in women than men for four aspects of life (looking after the home, looking after belongings, interpersonal interaction, social activities) (data not shown).

## Discussion

Participating in life situations "as and when you want" is an essential part of life, and establishing the natural history of person-perceived participation restriction in older adults provides a perspective on the effects of health on everyday life and the potential need for effective interventions to improve participation. Estimating incidence and recovery highlights the potential for participation restriction to be modified or prevented. We have previously reported that about one half of a general population sample of adults aged 50 and over report participation restriction at any one time; now in this prospective follow-up of these community-dwelling older adults, we have shown that there is a substantial degree of change in participation status over a three-year period. Nearly 30% of those who were participating "as and when they wanted" in all aspects of life at baseline indicated that they were not doing so in at least one aspect of life three years later, whereas almost one third of those who had a restriction at baseline were reporting at three-year follow-up that they were now free of restrictions in any aspect of their lives. In addition many of those who continue to indicate restriction in at least one aspect of life at three years, have indicated recovery and onset of restriction in different areas to those indicated at baseline. However these figures must be set in the context that, for most people, overall status remained unchanged; 39% of the sample remained restriction free at three year follow-up and most persons reporting a restriction at baseline were still restricted in at least one aspect of life three years later. Although the three-year interval may miss meaningful transition between participation and participation restriction [12,15], our findings still highlight that participation status over this time period changes for some adults and remains stable for others.

These overall figures conceal substantial contrasts in the patterns of change with age. The high likelihood of persistence at older ages combined with the age-related increase in the 3-year onset underlines the cumulative problem of participation restriction as people become older – exemplified by the declining likelihood of recovery as people reach the oldest ages. Both incidence and persistence are also higher in women, especially in women aged 80 years and over, where half of those without restriction at baseline indicated at least one aspect of life restricted by three year follow-up, and 90% of women in this age-group with a baseline restriction continued to indicate restriction at three-year follow-up.

Older adults who were already experiencing restriction in one aspect of life at baseline were more likely to indicate restriction in other areas at three year follow-up than those free of restriction. This indicates that once restriction is present in one area of life, this becomes a risk factor or risk marker for further areas of life to become restricted. By contrast there is evidence that restriction is reversible for some people, notably when it occurs in men and adults in the younger age range of our sample of over-50 year olds, and when it is an isolated problem in one aspect of life. However these rates of recovery may be over-estimated for each aspect of life because they will include individuals who no longer wish to participate in that aspect of life, either because it has become too difficult to maintain participation (in which case the extent of persistence will be under-estimated) or because they simply no longer need to participate in such areas. This may be particularly true for work, education and looking after dependents, where persistence was lowest and the proportion with participation restriction decreased with age [22].

The majority of the onset of restriction in mobility outside the home, managing money, work, education or social activities is experienced in those with no restriction in any other aspect of life and this indicates how the profile of participation restriction begins (data not shown). These aspects of life provide potential priority areas in which early interventions may prevent further restriction. In this model, the evidence presented here highlights again the prime importance of mobility outside the home because it is the most common form of restriction and problems with mobility are often the first function to become limited in the disability process [37].

The estimates of frequency of onset and persistence of participation restriction, adjusting simultaneously for age, gender, socio-economic status and physical and mental health, in persons who were subsequently lost to follow-up were similar to those observed for persons who completed the KAP at three years. By contrast clear differences in age, gender, socio-economic status and general health were observed between those included in the analysis and those who had died at follow-up. Although prevalence declined in the three-year period because of this selective attrition, the overall number of people with participation restriction in the sample followed from baseline to three years has increased because the number of people who have indicated onset of participation restriction is greater than the number who indicated recovery.

There is a possibility that recovery may also be over-estimated due to regression to the mean which occurs when apparent abnormalities on initial investigation have a high probability of being at the extreme end of an individual's normal range because of random variation [38] or responder bias (people who recover may be more likely to respond to a follow-up questionnaire). However participation restriction at baseline was higher in persons who subsequently did not respond and it seems unlikely that recovery estimates are substantially biased. Alternatively some of the changes may be due to measurement error rather than a true change in participation, although repeatability investigations had suggested that most items on the KAP are not subject to large responder variations in short-term reporting [38].

Patterns of incidence and persistence over time for individual aspects of life varied with age and gender and were not necessarily the same as those cross-sectional relationships observed at baseline between prevalence, age and gender [22]. For example, at baseline the prevalence of restricted self-care increased with age, whereas the persistence of restricted self-care was higher in the younger age-groups (50–59, 60–69, 70–79) than in those aged 80 and over. In addition to the limitations of this study discussed previously (attrition, measurement error and regression to the mean), small numbers of those restricted at baseline for some individual items (such as self-care) and the consequent unstable estimate of change, may explain these inconsistent relationships between age and restriction patterns over time in some aspects of life.

We have previously shown that person-perceived participation restriction is common in adults aged 50 years and over in the community but the follow-up study has highlighted the substantial degree of change in participation status that can occur in a three-year time period. The results indicate the potential for prevention and reduction in the level of restriction. Prevention of onset or early intervention to reverse isolated restriction could potentially reduce the risk of progression to other areas of restriction. Even for persons who have established restrictions, the observations of a significant rate of reversibility underlines the need to identify ways to enhance such improvement.

Our results reflect previous studies of the changes and extent of limitations in other forms of disability [11,12]. Deeg [15] reported that 53% of adults aged between 55 and 85 did not report limitations in climbing stairs, cutting toenails or using transportation over a six-year period. Barberger-Gateau and colleagues [14] reported that limitation in three definitions of disability (activities of daily life, instrumental activities of daily life, mobility and housework) persisted in around 50% of adults aged 65 and over who had the particular definition at baseline. For participation, the causes of the onset, persistence and recovery are likely to be numerous. Although medical conditions play a major role, and medical interventions may directly improve participation, environmental influences on functioning may be of greater importance in older adults, such that participation may be maintained even in the presence of health conditions, impairments and activity limitations [39]. In future analyses we will investigate the role of such influences on change in participation status.

## Conclusion

In this prospective follow-up study of community-dwelling older adults, person-perceived participation status changed during a three-year period, with onset of restriction occurring as well as persistence and recovery. The onset and persistence of any participation restriction was more common the older the age-group. However for some individual aspects, restriction decreased with age. The results highlight the potential for prevention and reduction in the level of restriction in older adults.

## Abbreviations

KAP: Keele Assessment of Participation; SF-12: Medical Outcomes Study Short Form 12 Questionnaire

## Competing interests

The authors declare that they have no competing interests.

## Funding

This study is supported financially by the Medical Research Council, UK (grant code: G9900220) and by the North Staffordshire Primary Care R&D Consortium.

## Authors' contributions

All authors contributed substantially to (i) the conception and design of the study, acquisition of data and analysis and interpretation of data, (ii) drafting of this manuscript and have given final approval of this version for publication.

## Supplementary Material

Additional file 1. Observed onset and persistence of restriction in any and each aspect of life at three years Click here for file
