# Supplementary material for: Onset and persistence of person-perceived participation restriction in older adults: a 3-year follow-up study in the general population
Source: Health Qual Life Outcomes. 2008 Nov 5;6:92. doi: 10.1186/1477-7525-6-92 (PMC2613375; doi:10.1186/1477-7525-6-92)
Supplement: Additional file 1. — Observed onset and persistence of restriction in any and each aspect of life at three years [file 1477-7525-6-92-S1.doc]

Additional Data Files

Table A – Observed onset and persistence of any restriction at three-year follow-up in older adults who completed the KAP at baseline and three-year follow-up; overall and stratified by age and gender

|  | **Onset of any restriction**  **(n=2049)**  **Freq (%)** | **Persistence of any restriction**  **(n=1660)**  **Freq (%)** |
| --- | --- | --- |
| Overall | 599 (29.2) | 1149 (69.2) |
| Gender Females  Males | 305 (28.4)  294 (30.2) | 721 (72.8)  428 (63.9) |
| Age group  Overall  50-59 years  60-69 years  70-79 years  80+ yrs  Females  50-59 years  60-69 years  70-79 years  80+ yrs  Males  50-59 years  60-69 years  70-79 years  80+ yrs | 220 (26.1)  200 (27.4)  146 (36.5)  33 (44.6)  117 (25.4)  97 (25.9)  73 (36.3)  18 (47.4)  103 (26.9)  103 (28.9)  73 (36.7)  15 (41.7) | 380 (64.6)  352 (66.0)  298 (74.3)  119 (86.2)  229 (67.6)  211 (71.0)  192 (75.6)  89 (89.0)  151 (60.6)  141 (59.8)  106 (72.1)  30 (79.0) |

## Table B – Observed onset and persistence of participation restriction in individual aspects of life at three years, in older adults who completed the KAP at baseline and three-year follow-up

|  | **Mobility within the home**  **Freq (%)** | **Mobility outside the home**  **Freq (%)** | **Self-care**  **Freq (%)** | **Looking after the home**  **Freq (%)** | **Looking after belongings**  **Freq (%)** | **Looking after dependents**  **Freq (%)** | **Interpersonal interaction**  **Freq (%)** | **Managing money**  **Freq (%)** | **Work**  **Freq (%)** | **Education**  **Freq (%)** | **Social activities**  **Freq (%)** |
| --- | --- | --- | --- | --- | --- | --- | --- | --- | --- | --- | --- |
| Onset of restriction  Overall  50-59 years  60-69 years  70-79 years  80+ years | 186 (5.6)  50 (3.8)  51 (4.5)  58 (8.2)  27 (16.2) | 341 (11.5)  86 (6.9)  100 (9.78)  115 (19.5)  40 (32.8) | 211 (6.1)  57 (4.2)  70 (5.9)  57 (7.7)  27 (15.2) | 260 (8.0)  92 (7.3)  73 (6.5)  73 (10.6)  22 (13.8) | 180 (5.4)  65 (5.0)  52 (4.5)  46 (6.4)  17 (9.7) | 138 (3.9)  61 (4.6)  53 (4.4)  21 (2.7)  3 (1.5) | 248 (7.6)  74 (5.8)  66 (5.8)  72 (10.5)  36 (21.1) | 333 (10.5)  109 (8.8)  121 (11.1)  83 (12.6)  20 (11.8) | 113 (3.2)  51 (3.7)  44 (3.6)  17 (2.2)  1 (0.5) | 130 (3.7)  78 (5.9)  36 (3.0)  14 (1.8)  2 (1.0) | 311 (9.8)  110 (8.9)  99 (9.0)  80 (11.8)  22 (12.8) |
| Persistence of restriction  Overall | 171 (44.8) | 464 (63.2) | 99 (38.1) | 232 (48.6) | 151 (42.9) | 45 (21.6) | 223 (51.6) | 263 (48.8) | 19 (12.6) | 40 (21.5) | 180 (33.9) |

**Table C**. **Associations between the onset of restriction in individual aspects of life and amount of restriction at baseline in older adults who completed the KAP at baseline and three-year follow-up; unadjusted, adjusted for gender and adjusted for age; Odds ratios and 95% confidence intervals**

|  | Unadjusted  OR* (95% CI) ^†^ | Gender adjusted  Adj OR (95% CI) | Age adjusted  Adj OR (95% CI) |
| --- | --- | --- | --- |
| Mobility within the home  1-2  3+ | 2.90 (2.04, 4.12)  7.98 (5.36, 11.89) | 2.92 (2.05, 4.15)  8.11 (5.44, 12.09) | 2.74 (1.93, 3.91)  7.39 (4.94, 11.05) |
| Mobility outside the home  1-2  3+ | 2.00 (1.56, 2.55)  3.78 (2.48, 5.76) | 1.98 (1.55, 2.52)  3.73 (2.45, 5.69) | 1.98 (1.54, 2.53)  4.17 (2.69, 6.45) |
| Self-care  1-2  3+ | 2.96 (2.08, 4.19)  8.40 (5.83, 12.10) | 2.97 (2.09, 4.21)  8.51 (5.90, 12.28) | 2.85 (2.01, 4.05)  7.99 (5.54, 11.53) |
| Looking after the home  1-2  3+ | 2.24 (1.69, 2.97)  5.82 (4.01, 8.46) | 2.23 (1.68, 2.96)  5.80 (3.99, 8.43) | 2.17 (1.63, 2.88)  5.58 (3.83, 8.12) |
| Looking after belongings  1-2  3+ | 2.63 (1.83, 3.76)  8.18 (5.50, 12.17) | 2.61 (1.82, 3.74)  8.10 (5.44, 12.07) | 2.58 (1.80, 3.70)  7.93 (5.31, 11.83) |
| Looking after dependents  1-2  3+ | 1.25 (0.82, 1.91)  2.96 (1.97, 4.44) | 1.24 (0.81, 1.90)  2.90 (1.93, 4.37) | 1.32 (0.87, 2.02)  3.32 (2.20, 5.02) |
| Interpersonal interaction  1-2  3+ | 2.61 (1.94, 3.51)  6.38 (4.45, 9.15) | 2.58 (1.92, 3.48)  6.23 (4.34, 8.93) | 2.48 (1.84, 3.34)  6.00 (4.17, 8.63) |
| Managing money  1-2  3+ | 1.65 (1.27, 2.14)  1.99 (1.44, 2.75) | 1.64 (1.26, 2.12)  1.97 (1.43, 2.73) | 1.62 (1.25, 2.11)  1.94 (1.40, 2.68) |
| Work  1-2  3+ | 1.37 (0.90, 2.08)  1.18 (0.69, 2.01) | 1.36 (0.89, 2.07)  1.16 (0.68, 1.99) | 1.44 (0.94, 2.19)  1.28 (0.75, 2.21) |
| Education  1-2  3+ | 1.33 (0.91, 1.94)  0.60 (0.32, 1.12) | 1.33 (0.90, 1.94)  0.60 (0.32, 1.12) | 1.48 (1.01, 2.17)  0.69 (0.37, 1.29) |
| Social activities  1-2  3+ | 1.75 (1.34, 2.28)  2.07 (1.48, 2.90) | 1.75 (1.34, 2.28)  2.07 (1.48, 2.90) | 1.71 (1.31, 2.24)  2.00 (1.43, 2.81) |

* Odds ratio; ^†^ 95 percent confidence interval
